# Research advances of Sappanone A in inflammation-related diseases

**DOI:** 10.3389/fmed.2025.1569732

**Published:** 2025-05-08

**Authors:** Jie Fu, Xiu Chen, Jinglun Li, Lilei Peng

**Affiliations:** ^1^Department of Neurology, The Affiliated Hospital of Southwest Medical University, Luzhou, China; ^2^Department of Neurosurgery, The Affiliated Hospital of Southwest Medical University, Luzhou, China

**Keywords:** Sappanone A, inflammation, antioxidant, molecular mechanism, inflammation-related diseases

## Abstract

Sappanone A (SA), a kind of homoisoflavanone extracted from the dry heartwood of *Caesalpinia sappan L.*, has been shown to possess diverse bioactivities involving anti-inflammatory, antioxidant, and anti-apoptotic properties. Sustained proinflammatory state is a major factor in the occurrence and development of various diseases. Given the characteristics of SA, many studies have explored the effect of SA on inflammation-related diseases, which uncovered the multifaceted therapeutic potential of SA in such diseases. In this mini-review, we summarized the current achievements of SA on inflammation-related diseases (such as myocardial ischemia-reperfusion injury, liver injury, respiratory diseases, and kidney injury, etc.), in order to provide useful insights into the role of SA in inflammation-related diseases and benefit future clinical applications.

## 1 Introduction

Inflammation is a biological response of the immune system to stimuli such as pathogens, cellular injury, and irritants. A proper inflammatory response protects our body against these harmful stimuli and initiates the recovery process (1). However, uncontrolled inflammation could result in tissue and organ damage, contributing to the pathogenesis of various diseases (2). Targeting inflammatory cascade may be an effective strategy for such diseases, but drugs currently used to treat inflammation-related diseases are often associated with adverse effects, including gastrointestinal discomfort, anaphylaxis, pharmacoresistance, hematopoietic toxicity, and nephrotoxicity (3). Therefore, there is a great need to develop effective and safe anti-inflammatory drugs. In recent years, a wide range of natural products have demonstrated anti-inflammatory properties with fewer side effects, making them potential candidates for the treatment of inflammation-related diseases (4, 5).

Sappanone A (SA) (chemical formula: C_16_H_12_O_5_), a kind of homoisoflavanone derived from the dry heartwood of *Caesalpinia sappan L.*, has been suggested to exert diverse biological activities involving anti-oxidation, anti-inflammation, and anti-apoptosis (6). Recently, the role of SA in inflammation-related diseases has gained increasing attention. The mechanisms by which SA alleviates inflammation-related conditions may involve its regulation of inflammatory mediators, macrophage polarization, oxidative stress, and cell apoptosis, etc. Pro-inflammatory mediators can initiate the inflammatory cascades, and thus aggravate tissue injuries (7). Studies have indicated that SA can prevent the generation of pro-inflammatory cytokines including tumor necrosis factor-α (TNF-α), interleukin-6 (IL-6) and IL-1β, and alleviate inflammatory responses (8). Macrophage polarization plays a critical role in the development and progression of inflammatory reactions (9). Macrophages are polarized into pro-inflammatory M1-like macrophages (M1) or anti-inflammatory M2-like macrophages (M2) under diverse inflammatory circumstances (10). M1 macrophages release pro-inflammatory factors to sustain proinflammatory responses, while M2 macrophages exert anti-inflammatory effects to repair injured tissues (11). When macrophage polarization is disrupted, it leads to an imbalance in the M1/M2 ratio, which exacerbates inflammatory response and participates in the pathogenesis of various inflammatory conditions (10). Notably, SA has been reported to have a regulatory effect on macrophage polarization. In detail, SA could inhibit M1 polarization of macrophages and promote M2 polarization, thereby reducing hepatic inflammation and mitigating the severity of liver fibrosis (12). Additionally, oxidative stress is another factor that aggravates tissue damage under the inflammatory conditions. It is suggested that SA exerts antioxidant effects by inhibiting the production of oxidative stress markers such as malondialdehyde (MDA) and myeloperoxidase (MPO), and by enhancing the activity of antioxidant enzymes such as superoxide dismutase (SOD) and glutathione peroxidase (GSH-Px) (13). Both oxidative stress and inflammation induce cell apoptosis, which eventually leads to tissue injury. Interestingly, the anti-apoptotic effect of SA has been suggested against cerebral ischemia/reperfusion injury, via attenuating excessive endoplasmic reticulum stress (14). Despite the increasing interest in SA, a comprehensive and up-to-date review of its molecular mechanisms against inflammation-related diseases is still lacking. Therefore, this mini-review aims to summarize current knowledge on the therapeutic effects and the potential mechanisms of SA in inflammation-related diseases, providing useful insights for future research.

## 2 Research progress of SA in inflammation-related diseases

Due to the significant anti-inflammatory, anti-oxidative, and anti-apoptotic effects, SA has been investigated for its potential to treat myocardial ischemia-reperfusion injury, liver injury, respiratory diseases, and other inflammation-related conditions including kidney injury, neuroinflammation, psoriasis, rheumatoid arthritis, and osteoarthritis (Table 1).

**TABLE 1:** Mechanisms and regulation of Sappanone A for the treatment of inflammation-related diseases.

Inflammation-related diseases	Mode of study	Animal model/cell lines	Dose	Target	Effect	References
Myocardial ischemia- reperfusion injury	*In vivo*	SD rats	50 mg/kg for 5 days	Tgfb1, Tgfb2, Cd4, Cd8a, Il18, Pik3cd, Tnfrsf1a, and Casp3	Reduced inflammation and cell apoptosis, thus improving left ventricular dysfunction	(21)
	*Ex vivo*	Wistar rats	10, 20 and 40 mg/kg pretreatment for 1 h	Keap1/Nrf2 signaling pathway	Inhibited myocardial apoptosis and oxidative stress, and ameliorated cardiac function	(19)
	*In vitro*	H9c2 cells	25 μM pretreatment for 1 h	Mitochondrial apoptosis and PI3K-Akt-Gsk-3β signaling pathway	Inhibited cardiomyocyte apoptosis	(6)
	*Ex vivo*	Wistar rats	10, 100, 1,000 μM for the first 15 min of reperfusion after ischemia	AMPK	Reduced mitochondrial dysfunction and myocardial injury	(25)
Liver injury	*In vivo*/*in vitro*	C57BL/6N mice and RAW 264.7 cells	50 mg/kg at 1 h and 13 h after LPS administration (*in vivo*), 100 μÌ for 24 h (*in vitro*)	Fbf1 and macrophage polarization	Reduced inflammation, and promoted the apoptosis of injured hepatocytes, thus ameliorating LPS-induced acute liver injury	(32)
	*In vivo*/*in vitro*	C57BL/6 N mice and primary murine hepatocytes	25, 50, 100 mg/kg for 7 days (*in vivo*), 5, 20, 50 μM pretreatment for 30 min and 1 h (*in vitro*)	Nrf2	Reduced hepatic cell death, inflammatory response and oxidative stress, thus ameliorating acetaminophen-induced acute liver injury	(28)
	*In vivo*/*in vitro*	C57BL/6 mice, Hep3B cells and Hepa1-6 cells	20 mg/kg for 14 days (*in vivo*), 5, 10, 20 and 40 μM for 24 h (*in vitro*)	IMPDH2 and NRF2/xCT/GPX4	Induced ferroptosis and inhibited the growth of hepatocellular carcinoma	(37)
	*In vivo*/*in vitro*	C57BL/6N mice, AML12 cells, RAW 264.7 cells, and human HSC line	25, 50, and 100 mg/kg for 4 weeks (*in vivo*), 1, 10 and 100 μM for 24 h (*in vitro*)	PPARγ-mediated macrophage polarization	Reduced inflammatory response, oxidative stress and hepatocyte apoptosis, and ameliorated carbon tetrachloride-induced liver fibrosis	(12)
	*In vivo*/*in vitro*	C57BL/6 mice and Bone marrow-derived macrophages	50 mg/kg at 3 h before and 1 h after concanavalin A administration (*in vivo*), 2, 4 and 8 μM pretreatment for 3 h (*in vitro*)	NF-κB and MAPK signaling pathways	Inhibited M1 macrophage polarization and inflammatory response, thus reducing concanavalin A-induced liver injury	(31)
Respiratory diseases	*In vivo*/*in vitro*	ICR mice and RAW264.7 cells	50, 100 mg/kg (compound 6o), 2.5, 5, 10, 250, 500 and 1,000 μM (compounds 6n-6r)	PDE4 enzyme	Reduced inflammation and oxidative stress, exhibiting therapeutic potential for the treatment of COPD	(46)*
	*In vivo*/*in vitro*	ICR mice and RAW 264.7 cells	50, 100 mg/kg for 7 days (*in vivo*), 10, 20 and 40 μM pretreatment for 1 h (*in vitro*)	PDE4 enzyme	Reduced inflammation and oxidative stress, preventing LPS-induced acute lung injury	(45)
	*In vivo*	BALB/c mice	12.5, 25 and 50 mg/kg pretreatment for 1 h	Nrf2/HO-1 signaling pathway	Attenuated the airway inflammation and mucus hypersecretion, and inhibited ovalbumin-induced asthma	(49)
	*In vivo*/*in vitro*	C57BL/6 mice and BEAS-2B cell line	20, 40 mg/kg pretreatment for 1 h (*in vivo*), 5, 10, 20 μM pretreatment for 1 h (*in vitro*)	NF-κB signaling pathway	Reduced inflammation and ameliorated LPS-induced acute lung injury	(40)
Kidney injury	*In vivo*/*in vitro*	BALB/c mice and HK-2 cells	10, 20, 40 mg/kg for 3 days (*in vivo*), 10, 20, 30 μM pretreatment for 1 h (*in vitro*)	Nrf2 and NF-κB signaling pathways	Reduced inflammation and oxidative stress, thus improving cisplatin-induced kidney injury	(13)
	*In vivo*/*in vitro*	C57BL/6J mice and SV40-MES-13 cells	10, 20, and 30 mg/kg for 12 weeks (*in vivo*), 10, 20, and 30 μM pretreatment for 1 h (*in vitro*)	NF-κB signaling pathway	Inhibited diabetes-induced kidney inflammation and fibrosis	(53)
Neuroinflammation	*In vivo*/*in vitro*	BALB/c mice, BV-2 cells, and primary microglia	50, 100 mg/kg pretreatment for 1 h (*in vivo*), 5, 10, 20 μM for 30 min, 1, 3, 4, 6, 8, 12, or 24 h (*in vitro*)	IMPDH2	Suppressed the production of inflammatory cytokines and reduced microglia-mediated neuroinflammation	(54)
	*In vivo*/*in vitro*	SD rats and PC12 cell line	10, 20, 40 mg/kg for 3 days (*in vivo*), 10, 20, and 30 μM for 24 h (*in vitro*)	Endoplasmic reticulum stress	Inhibited neuroinflammation, oxidative stress and cell apoptosis, thus alleviating cerebral ischemia-reperfusion injury	(14)
Psoriasis	*In vivo*/*in vitro*	BALB/c mice and HaCaT cells	10, 20, 40 mg/kg for 6 days (*in vivo*), 10, 20, 40 μM pretreatment for 2 h (*in vitro*)	MMP 8 and IL-17	Inhibited inflammation and excessive proliferation of keratinocytes, and thus alleviated psoriasis-like skin lesions	(57)
Rheumatoid arthritis	*In vivo*/*in vitro*	Wistar rats and HFLS-RA cells	25, 50 mg/kg for 10 days (*in vivo*), 10, 20, 40 μM for 24 h (*in vitro*)	PI3K/AKT/NF-κB and JAK2/STAT3 signaling pathways	Inhibited inflammation and bone destruction	(61)
Osteoarthritis	*In vivo*/*in vitro*	SD rats and primary chondrocytes	20, 50 mg/kg twice a week (*in vivo*), 5, 10, 20 μM for 24 h (*in vitro*)	SIRT1/Nrf2 signaling pathway	Inhibited inflammatory response and extracellular matrix degradation, and alleviated chondrocyte ferroptosis	(66)

Annotation: *derivatives of Sappanone A.

### 2.1 SA and myocardial ischemia-reperfusion injury

Acute myocardial infarction (AMI) is one of the leading causes of mortality worldwide (15). Early and efficient myocardial reperfusion treatment is the main therapeutic strategy for AMI, particularly in cases of ST-segment elevation (16). However, following the myocardial reperfusion therapy, the rapid restoration of blood supply to the ischemic myocardium may exacerbate myocardial damage, which is known as myocardial ischemia-reperfusion injury (MIRI) (17). The underlying mechanism of MIRI may involve multiple pathophysiological processes, including inflammation, oxidative stress-mediated mitochondrial dysfunction, and apoptosis (18). Recently, several investigations have been carried out to explore the effect of SA on MIRI and the related mechanisms.

Shi et al. (19) reported that intraperitoneal pretreatment with 20 mg/kg SA one hour prior to MIRI model induction reduced myocardial infarct size and promoted cardiac function recovery in isolated Langendorff hearts, indicating cardioprotective properties of SA. The potential mechanism may be associated with SA’s anti-oxidant activity. SA administration reduced the levels of reactive oxygen species (ROS) and MDA, and increased the activities of GSH-Px and SOD in ischemic myocardium through induction of nuclear factor E2-associated factor 2 (Nrf2) nuclear accumulation, activation of Nrf2 transcription, and upregulation of expression levels of Nrf2 target genes. Aside from its impact on oxidative stress, SA has been observed to regulate cell apoptosis and thus prevent cardiomyocyte injury. The phosphoinositide 3-kinase (PI3K)/protein kinase B (AKT) pathway is an important signaling pathway involved in cell apoptosis, and activation of the PI3K-Akt pathway blocks apoptosis by inhibiting pro-apoptotic gene expression and promoting anti-apoptotic gene expression (20). SA demonstrated the ability to mitigate cardiomyocyte injury and apoptosis induced by hypoxia/reoxygenation through activating the PI3K/AKT/glycogen synthase kinase-3β (GSK3β) signaling pathway (6). In line with these *ex vivo* or *in vitro* experiments, an *in vivo* study from Jo et al. (21) also confirmed cardiovascular protective effects of SA, and they found that oral administration of SA reduced myocardial infarct size and improved left ventricular (LV) dysfunction in a rat myocardial ischemia/reperfusion injury model. In this study, although SA failed to significantly improve the LV systolic function, it remarkably reversed the ratio of transmitral Doppler early filling velocity to tissue Doppler early diastolic mitral annular velocity (E/E’), a parameter of LV diastolic function, which returned to almost normal levels after SA treatment. Furthermore, they also explored the underlying mechanisms, which may be associated with SA’s anti-inflammatory and anti-apoptotic effects by decreasing the expression of genes involved in inflammation (*Tgfb1*, *Tgfb2*, *Tnfrsf1a*, *Il18*, *Pik3cd*, *Cd4*, and *Cd8a*) as well as apoptosis (*Casp3*). Collectively, these findings suggest that the cardioprotective effects of SA may be attributable to its antioxidant, anti-inflammatory, and anti-apoptotic properties.

Of note, mitochondrial dysfunction plays a key role in the pathogenesis of MIRI. Decreased mitochondrial energy metabolism during cardiac ischemia could be restored following reperfusion, but the early mitochondrial calcium overload and excessive ROS production boost the opening of mitochondrial permeability transition pore (mPTP) and depolarization of mitochondrial membrane potential (MMP), ultimately leading to cell apoptosis (22). By restoring the mitochondrial homeostasis, medicinal plants or their bioactive extracts can exert a protective effect on MIRI (23, 24). Likewise, SA has also been reported to reduce MIRI by regulating mitochondria-related processes including mitochondrial apoptosis and mitochondrial quality control. In the *in vitro* study, SA pretreatment inhibited mitochondrial apoptosis pathway involving mPTP opening, loss of transmembrane potential (Δψm), and cleavage of mitochondrial apoptosis-related proteins (caspase-9 and caspase-3) (6). Moreover, in isolated Langendorff hearts, SA postconditioning has been validated to regulate mitochondrial quality control including promoting mitochondrial biogenesis, balancing mitochondrial dynamics and reinforcing phosphatase and tensin homolog (PTEN) induced kinase 1 (PINK1)/Parkin-dependent mitophagy via activating AMP-activated protein kinase (AMPK), thereby alleviating MIRI and mitochondrial dysfunction (25). Indeed, these *in vitro* or *ex vivo* studies offer valuable insights into SA-related regulations via targeting mitochondria in MIRI, but future *in vivo* investigations are still needed to confirm these findings.

### 2.2 SA and liver injury

As a primary organ for detoxification and metabolism, liver is highly susceptible to oxidative stress and inflammatory responses. Targeting these processes has been suggested as a potential strategy for treating liver injury (26). Previous studies have reported that SA protects against liver injury through regulating oxidative stress and inflammation. Acetaminophen (APAP)-induced liver injury is one of the leading causes of acute liver failure, and the underlying mechanism may be involved in dysregulated oxidative stress (27). Zhou et al. (28) investigated the effect of SA treatment on APAP-induced liver toxicity and observed that SA enhanced GSH synthesis and reduced the levels of MDA, thus reducing oxidative stress-related hepatotoxicity induced by APAP. Moreover, SA also decreased APAP-evoked hepatic inflammation through inhibiting the expression of inflammatory mediators such as TNFα and IL-1β. Furthermore, they found that Nrf2 mediated the protective effects of SA on APAP-induced hepatotoxicity, and inhibiting Nrf2 reversed the elevated GSH concentrations and reduced MDA levels by SA, thus blocking liver-protective effects of SA. It is worth mentioning that this study compared the therapeutic effect of SA with N-acetylcysteine (NAC) which is primarily used in preventing liver damage from APAP overdose, which indicated that SA had a similar protective impact to NAC, and the combinative administration of SA and NAC resulted in a more remarkable hepatoprotective effect than a single treatment. These results unveiled the critical role of SA in reducing liver injury and established a foundation for its future therapeutic applications, either alone or co-treatment with other drugs, in order to improve treatment outcomes.

In addition, it is shown that macrophage polarization exerts a critical role in the occurrence and development of various liver diseases (29). Macrophages mainly polarize to pro-inflammatory M1 and anti-inflammatory M2 phenotypes (11). Promoting polarization of M1-like inflammatory macrophages toward M2-like anti-inflammatory macrophages has been suggested to improve liver diseases mainly involved in inflammatory injury, such as acute liver injury and carbon tetrachloride (CCl4)-induced liver fibrosis (29). Notably, recent studies have indicated that SA could regulate macrophage polarization and thus reduce liver damage. Concanavalin A (Con A)-induced liver damage shares similar pathophysiological features with viral hepatitis and autoimmune liver disorders, which is one of the most commonly used animal models for exploring immune-related liver injury (30). A recent study from Yan et al. (31) reported that SA pretreatment remarkably reduced the architectural destruction of liver lobules, hepatocyte necrosis as well as inflammatory cell infiltration induced by Con A. Furthermore, they found that SA also inhibited pro-inflammatory M1 macrophage activation, and the proportion of M1 macrophage decreased from 57.6% to 46.3% in mice treated with Con A after SA pretreatment. The potential protective mechanism of SA may be partially involved in its inhibition of the mitogen-activated protein kinase (MAPK) and nuclear factor kappaB (NF-κB) signaling pathways. Additionally, in a lipopolysaccharide-induced septic liver injury mouse model, significant liver damage characterized by sporadic bleeding, infiltration of inflammatory cells and localized tissue necrosis was observed, but SA reversed such effects and promoted liver recovery by increasing damaged hepatocyte apoptosis through inhibiting fas binding factor 1 (FBF1) and modulating M1/M2 macrophage polarization (32). Liver fibrosis is a kind of abnormal wound healing response generated against the damage to the liver, which is the ultimate pathological result of multiple chronic hepatic diseases (33, 34). The pathogenesis of hepatic fibrosis is proposed to be associated with hepatic cell necroptosis, Kupffer cell activation and recruitment, hepatic stellate cell activation, hepatic inflammation, ROS production, and ROS-induced oxidative stress (12). Qi et al. (12) used CCl4 to establish an experimental mouse liver fibrosis model, and observed that SA improved CCl4-induced liver injury characterized by the destruction of hepatic lobule structure, disorderly arrangement of the hepatic cell cords, and inflammatory cell infiltration. Furthermore, they also found that SA mitigated CCl4-induced liver fibrosis by inhibiting oxidative stress-mediated hepatic cell death and triggering the polarization of macrophages toward an anti-inflammatory M2 phenotype via activating peroxisome proliferator-activated receptor gamma (PPARγ).

Besides, SA could also serve as an anti-tumor agent. Hepatocellular carcinoma (HCC), a primary liver malignancy, is the sixth most common cancer and fourth leading cause of malignancy-related death worldwide (35). Ferroptosis, a non-apoptotic cell death, could cause inflammatory responses, and inhibit the polarization of tumor-associated macrophages toward anti-inflammatory and protumoral (M2-like) states, which is a potential target for treating HCC (36). A recent study by Xing et al. (37) demonstrated that SA could enhance ferroptosis via the NRF2/cystine-glutamate antiporter (xCT)/glutathione peroxidase 4 (GPX4) pathway, and thus inhibit the growth of HCC both *in vitro* and *in vivo*. Furthermore, they found that inosine monophosphate dehydrogenase 2 (IMPDH2) was the critical pharmacological target, since knockdown of IMPDH2 reversed SA-induced ferroptosis in HCC cells.

Together, these results suggest that SA may be a promising hepatoprotective agent for the treatment of liver injury.

### 2.3 SA and respiratory diseases

Studies have suggested that SA holds great promise as a therapeutic agent for the treatment of both acute lung injury (ALI) and chronic respiratory diseases (CRD). ALI or acute respiratory distress syndrome (ARDS) is one of the most severe lung diseases, which is associated with high morbidity and mortality rates (38). It is well documented that excessive inflammatory response contributes to the development of ALI (39). Of note, an investigation from Du et al. (40) applied lipopolysaccharide to establish animal and cell models of ALI, and unveiled that SA administration dose-dependently decreased the mRNA levels of pro-inflammatory cytokines TNF-α, IL-6, and IL-1β, mitigated oxidative stress as well as suppressed epithelial apoptosis, which was mediated partly by inhibiting the activation of the NF-κB signaling pathway. CRD are a group of disorders affecting the airways and other lung components, mainly including chronic obstructive pulmonary disease (COPD) and asthma (41). The pathogenesis of CRD is complicated, which is closely associated with inflammation and oxidative stress (42). Emerging evidence indicates that natural products hold substantial potential in treating CRD (43, 44). In recent years, the effect of SA on CRD, especially COPD and asthma, has garnered increasing attention from researchers. The vicious circle of oxidative stress and inflammation is closely involved in the pathogenesis of COPD, and thus antioxidant and anti-inflammatory bifunctional drugs could block this vicious cycle and hold great potential for the treatment of COPD. Wang et al. (45) employed lipopolysaccharide to establish lung injury models and investigated the anti-inflammatory and antioxidant effects of SA. They found that SA could serve as a natural phosphodiesterase 4 (PDE4) inhibitor with dual anti-inflammatory and antioxidant activities to reduce lung injury via inhibiting the release of pro-inflammatory cytokines and the activity of oxidative enzymes. Therefore, they proposed that SA may be a promising drug for treating COPD. Additionally, they designed and synthesized 27 derivatives of SA, and screened out a series of compounds with superior anti-inflammatory activity (46). For instance, compound 6o was found to dose-dependently suppress lipopolysaccharide-induced TNF-α generation in both *in vivo* and *in vitro* models, and the inhibition percentage of TNF-α by compound 6o (100 mg/kg) *in vivo* reached 54.7%. Furthermore, they also observed that these compounds exhibited better free radical scavenging activity than edaravone. Hence, these candidate compounds may have enhanced therapeutic potential for treating COPD. Asthma is a chronic inflammatory lung disease, which is characterized by inflammatory cell infiltration, airway inflammation, and airway hyperreactivity (47, 48). Liu et al. (49) demonstrated that SA alleviated allergic airway inflammation by regulating the Th1/Th2 balance via activating the Nrf2/heme oxygenase-1 (HO-1) signaling pathway in a mouse model of ovalbumin-induced asthma. In summary, these studies highlight the promising potential of SA or its derivatives in the treatment of respiratory diseases.

### 2.4 SA and other inflammation-related diseases

Kidney injury is a common clinical kidney syndrome caused by various pathogenic factors, which is characterized by damaged renal function and the accumulation of metabolic waste and toxins in the body (50, 51). Inflammatory response and oxidative stress contribute to the pathogenesis of kidney injury (52). Cisplatin, a widely used anti-tumor drug, often induces nephrotoxicity due to increased inflammatory response and oxidative stress. SA has been shown to reduce the production of pro-inflammatory cytokines TNF-α and IL-1β by inhibiting NF-κB activation, and to increase the activities of GSH-Px and SOD by activating the Nrf2 signaling pathway, thereby alleviating cisplatin-induced renal injury and improving renal function (13). Additionally, NF-κB signaling pathway is also implicated in the protective effect of SA on diabetic kidney disease. Wang et al. (53) reported that SA inhibited kidney inflammation and fibrosis via decreasing the expression of NF-κB and enhancing the expression of total NF-κB inhibitor alpha (IκBα), thus preventing the progression of diabetic kidney disease.

SA also plays a critical role in mitigating neuroinflammation. It is reported that SA could reduce neuroinflammation, oxidative stress and cell apoptosis in cerebral ischemia-reperfusion injury, thereby ameliorating brain tissue damage, which may be involved in its inhibitory effect on excessive endoplasmic reticulum stress (14). Moreover, a study by Liao et al. (54) indicated that SA could directly bind to the conserved cysteine residue 140 (Cys140) of IMPDH2, and further lead to IMPDH2 inactivation, thereby suppressing neuroinflammatory responses. Moreover, they also observed that SA at a high dose (100 mg/kg) did not show obvious hematological adverse effects compared to the same dose of mycophenolic acid (MPA), exhibiting good drug safety, which may be associated with SA specifically targeting IMPDH2 while not IMPDH1.

As the first barrier of the human body, the skin senses diverse stimuli and helps maintain the stability of the body’s internal environment (55). When the skin’s barrier function is damaged, skin-related disorders may occur. Psoriasis is a chronic immune-mediated inflammatory skin disease characterized by abnormal keratinocyte proliferation and immune cell infiltration (56). A study from Li et al. (57) demonstrated that SA displayed anti-proliferation and anti-inflammatory effects via suppressing the matrix metalloproteinase 8 (MMP8) expression and IL-17 signaling pathway activation, thereby reducing imiquimod-induced psoriasis-like skin lesions. Thus, SA may be a novel promising anti-psoriasis drug.

Rheumatoid arthritis (RA) is a chronic systemic autoimmune disorder characterized by progressive joint inflammation and damage (58). Inflammatory signaling pathways have been indicated to play a critical role in the pathogenesis of RA (59, 60). Deng et al. (61) employed rats with collagen-induced arthritis (CIA) and human fibroblast-like synoviocytes (HFLS) models to investigate the effect and mechanism of SA on RA. They found that SA alleviated inflammatory symptoms and bone damage in CIA rats, and the underlying mechanism may be involved in inhibiting the production of pro-inflammatory cytokines (TNF-α, IL-1β, IL-6, and IL-17A) and promoting the release of anti-inflammatory cytokine (IL-10) via blocking the activation of PI3K/AKT/NF-κB and Janus kinase 2 (JAK2)/signal transducer and activator of transcription 3 (STAT3) signaling pathways. Furthermore, studies have demonstrated that HFLS exert tumor-like cellular behaviors, such as abnormal proliferation, migration/invasion into the blood and joints, as well as generation of inflammatory cytokines, which mainly contribute to synovial inflammation and persistent bone damage, thereby exacerbating the progression of RA (62, 63). Deng et al. (61) also observed that SA could inhibit the proliferation of HFLS and osteoclast activity, thus reducing joint destruction. Given the anti-inflammatory property and inhibitory effect on the unrestricted proliferation of HFLS, SA may become a candidate drug for RA treatment.

Osteoarthritis (OA) is a common degenerative joint disease characterized by articular cartilage damage, synovial inflammation, subchondral bone remodeling, and chronic pain (64). Abnormal oxidative stress and inflammatory responses contribute to the development of OA (65). A recent study revealed that SA inhibited inflammation and extracellular matrix degradation, and thus slowed down OA progression (66). Mechanistically, SA reduced chondrocyte ferroptosis via activating the Sirtuin 1 (SIRT1)/Nrf2 signaling pathway. Overall, these findings highlight the therapeutic potential of SA for OA.

## 3 Discussion and prospect

**FIGURE 1:**
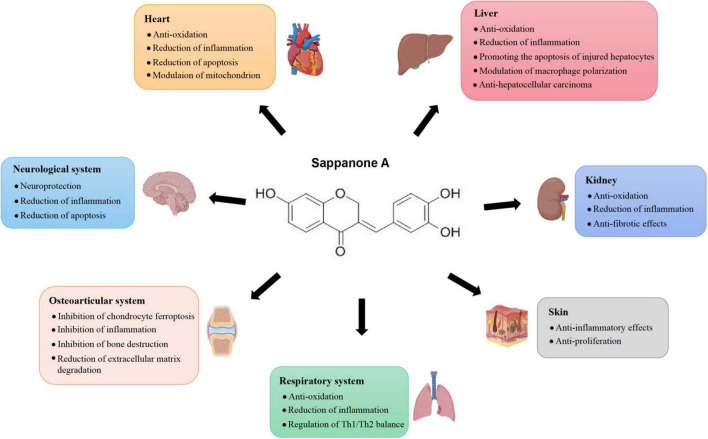
Effects of Sappanone A on different tissues and organs.

As a natural homoisoflavonoid, SA holds remarkable anti-inflammatory activities, and has been indicated as a potential candidate drug for treating various inflammation-related diseases including myocardial ischemia-reperfusion injury, liver injury, respiratory diseases, kidney injury, neuroinflammation, psoriasis, rheumatoid arthritis, and osteoarthritis. Based on the current publications, the protective role of SA in inflammation-related conditions could be attributed to its effects on inflammatory signaling pathways, oxidative stress, cell apoptosis, mitochondrial function, macrophage polarization and ferroptosis (Figure 1). SA could suppress the expression of pro-inflammatory mediators by preventing the activation of inflammatory signaling pathways and regulating macrophage polarization. Additionally, SA could modulate oxidative stress by targeting the Nrf2 signaling pathway and restoring mitochondrial homeostasis, thereby mitigating cell injury and apoptosis induced by oxidative stress. Furthermore, SA could exert anti-tumor effects by triggering ferroptosis of carcinoma cells, thus inhibiting the growth of carcinoma. These findings emphasize SA’s critical role in inflammation-related diseases and pave the way for its therapeutic applications in various disease contexts.

Despite their efficacy, the clinical application of drugs is often limited by their adverse effects. Fortunately, the current studies conducted *in vitro* or in animal models have indicated the good safety of SA. The study by Yan et al. (31) demonstrated that SA alone did not exhibit any adverse effects on the liver. Likewise, another investigation from Du et al. (40) found that chronic administration of 40 mg/kg SA showed no apparent harmful effects on the heart, kidneys, liver, or lungs of mice. Collectively, these studies suggested the favorable safety profile of SA, making it a promising candidate for clinical use.

According to the latest research evidence, as a naturally extracted product, SA possesses broad clinical application prospects. However, in order to progress to clinical applications, researchers should investigate: (1) Optimal dosages and safety of SA for administration to human inflammation-related diseases: the published studies conducted *in vitro* or in animal models have demonstrated the good efficacy and safety of SA in various inflammation-related diseases, but preclinical and clinical trials are needed to investigate the efficacy and safety of SA for administration to human patients; (2) Combination therapy approaches: some drugs currently used for inflammation-related diseases may have limited efficacy and narrow treatment window, such as NAC, and studies have indicated the stronger therapeutic effect of co-treatment of SA and NAC than monotherapy with NAC, which provided a novel insight into the clinical treatment strategies; (3) Developing targeted therapies: precise therapeutic interventions targeting specific genetic mechanisms responsible for inflammation-related diseases could be developed to enhance treatment effect and minimize side effects.
